# MALNUTRITION AND SARCOPENIA IN INPATIENT REHABILITATION: PREVALENCE AND ASSOCIATIONS WITH CHANGES IN BODYWEIGHT, MUSCLE STRENGTH, AND FUNCTIONAL INDEPENDENCE

**DOI:** 10.2340/jrm.v57.42215

**Published:** 2025-02-25

**Authors:** Undine LEHMANN, Katja UHLMANN, André MEICHTRY, Marc SPIELMANNS, Sabine SPIELMANNS, Ramin KHATAMI, Laura MARTI, Susanne RÜEGSEGGER, Reto W. KRESSIG, Caroline M. KISS, Clare MAGUIRE, Andrea ZURFLUH, Thimo MARCIN

**Affiliations:** 1 Division of Nutrition and Dietetics, Department of Health Professions, Bern University of Applied Sciences, Bern, Switzerland; 2 Zürcher RehaZentren | Klinik Wald, Wald, Switzerland; 3 Faculty of Health, Department of Pneumology, University of Witten-Herdecke, Germany; 4 Klinik Barmelweid AG, Barmelweid, Switzerland; 5 Department of Neurology, University Hospital Bern, Switzerland; 6 University Department of Geriatric Medicine Felix Platter, Basel, Switzerland; 7 University of Basel, Basel, Switzerland; 8 REHAB Basel Clinic for Neurorehabilitation and Paraplegiology, Basel, Switzerland; 9 Berner Reha Zentrum, Rehabilitation & Sports Medicine, Insel Group, University Hospital Bern, Bern, Switzerland

**Keywords:** malnutrition, sarcopenia, rehabilitation, prevalence, muscle strength, nutrition therapy

## Abstract

**Objective:**

To investigate the prevalence of malnutrition and sarcopenia in different disciplines of inpatient rehabilitation and the course of nutritional status parameters.

**Design:**

Multi-centre cross-sectional prevalence study and longitudinal observational study.

**Subjects/Patients:**

Inpatients (> 18 years) in geriatric, pulmonary, cardiovascular, internal medicine/oncological, musculoskeletal, or neurological rehabilitation in 5 rehabilitation centres were included.

**Methods:**

Malnutrition was assessed according to the Global Leadership Initiative on Malnutrition criteria. Sarcopenia was assessed according to the European Working Group on Sarcopenia in Older People criteria. Bodyweight, hand grip strength (HGS), and functional independence measure (FIM) were assessed within 3 days of admission and after 21 days of rehabilitation and analysed using linear mixed models with time*diagnosis interaction.

**Results:**

The study included 558 patients (51.8% male, median age 73.0 years). The overall prevalence of malnutrition and sarcopenia was 35.5% (95% CI 31.5, 39.6%) and 32.7% (95% CI 28.8, 36.8%), respectively. Patients with risk of malnutrition lost on average 1.14 kg (95% CI –1.64, –0.63) during rehabilitation. Patients slightly increased their HGS and FIM, irrespective of risk or diagnosis of malnutrition or sarcopenia. However, at the end of the rehabilitation, malnourished or sarcopenic patients had still a significantly lower bodyweight, HGS, and FIM than patients without (*p* < 0.01). Some 37.3% of patients at risk of and 35.4% with diagnosed malnutrition did not receive group or individual nutritional counselling.

**Conclusion:**

Malnutrition and sarcopenia are highly prevalent during inpatient rehabilitation. Nevertheless, dietitians are often not involved in the therapy. While nutritional parameters and functional independence improve, patients with malnutrition and sarcopenia remain on a lower level after 3 weeks of rehabilitation. Long-term follow-up after rehabilitation is recommended to prevent nutritional and muscular decline and related negative health outcomes.

## LAY ABSTRACT

Malnutrition that develops when the body does not get sufficient energy and nutrients, and sarcopenia, a disease resulting in muscle loss and function, are serious health concerns that can lead to negative consequences. We aimed to assess how frequent these problems are in various rehabilitation disciplines and how measures related to malnutrition and sarcopenia change during the course of a rehabilitation stay over 21 days. On average, almost half of the patients in our study suffered from malnutrition, sarcopenia, or both. Although rehabilitation slightly improved muscle strength and independence in daily activities, a considerable number of the patients left rehabilitation in a condition requiring long-term nutritional support. The study should raise awareness regarding the importance of nutritional management during and after rehabilitation.

Malnutrition is a common health concern in several populations, particularly in older adults. The associations between malnutrition and negative outcomes such as higher complication rates, increased length of hospital stay, increased morbidity, mortality, and related higher healthcare costs are well described (1–4).

The prevalence of malnutrition in the rehabilitation setting ranges from 14–65% worldwide (5). The prevalence data on risk and diagnosed malnutrition in the rehabilitation setting across various ages and rehabilitation disciplines are sparse. A multinational retrospective pooled analysis from the year 2010 found a higher prevalence of malnutrition in the rehabilitation setting (50%) compared with hospital (39%) or nursing home (14%) settings (6).

Malnutrition and sarcopenia frequently co-occur (7). Sarcopenia, a generalized skeletal muscle disorder, is associated with higher risk of falls, disability, and reduced quality of life (8). In a study involving older adults, it was found that malnutrition is a strong predictor of sarcopenia. Malnourished older adults had a 3–4 times higher risk of developing sarcopenia within 4 years compared with those who were well-nourished (9).

A systematic review investigating sarcopenia prevalence in inpatient rehabilitation concluded that sarcopenia might affect half of the patients in rehabilitation but that data are scarce (10). While there are some data on probable and diagnosed sarcopenia prevalence in geriatric rehabilitation (11, 12), data across different rehabilitation disciplines are limited.

Furthermore, few studies have evaluated the impact of rehabilitation on parameters related to malnutrition and sarcopenia such as changes in weight, muscle strength, or functioning. Moreover, there are inconsistent results related to responses to rehabilitation interventions in patients suffering from malnutrition and/or sarcopenia (13, 14).

In view of the limited amount of data, the current study aimed to (*i*) fill the gap and investigate the prevalence of malnutrition and sarcopenia in the rehabilitation setting on admission across rehabilitation disciplines and (*ii*) to assess the evolution of selected parameters linked to malnutrition and sarcopenia during inpatient rehabilitation.

## Methods

### Study design and participants

A multi-centre cross-sectional prevalence study and a longitudinal observational study were conducted in 5 rehabilitation centres in the German-speaking part of Switzerland from November 2022 until April 2023. Female and male adults ≥ 18 years old, treated in the following 6 disciplines of rehabilitation, were included: geriatric, pulmonary, cardiovascular, neurological, musculoskeletal, internal medicine/oncological (both disciplines combined) rehabilitation. The indication for rehabilitation in the respective disciplines is described in the Swiss rehabilitation rate reimbursement documents “ST Reha” (15). As an example, geriatric rehabilitation is indicated following acute illness or accident or chronic progressive multimorbidity with potentially reversible deterioration of functional abilities or impending loss of independence in basal and/or extended activities of daily living. Patients, usually above 75 years, with multimorbidity and presence of at least 2 of the listed geriatric syndromes are transferred to this rehabilitation (15). To ensure equal patient inclusion per discipline, patients were consecutively recruited until the pre-defined sample size for the respective discipline and centre (between 80 and 120 patients per discipline) was achieved.

Exclusion criteria were inability to give informed consent or to follow study procedures (e.g., due to delirium), an estimated life expectancy < 3 months and/or palliative care, isolation (e.g., due to COVID-19 infection), severe dehydration/volume overload (e.g., by visible oedema) or any other medical conditions that would limit execution or validity of a bioelectrical impedance analysis (BIA).

On admission, all patients in the defined disciplines were checked for eligibility. In the case of consent, screenings for malnutrition and sarcopenia were performed within 72 h upon admission. Following positive screening, assessments were conducted within 5 (or if not feasible within 7) days after admission. Patient characteristics such as age, height, weight, polypharmacy (5 or more medications at entry), previous stay in intensive care unit (ICU), comorbidities, and the Functional Independence Measure (FIM) were captured on admission.

For the secondary objective, longitudinal monitoring in the form of a change measure between admission (T1) and after 3 weeks of rehabilitation or within 3 days before discharge if discharge occurs first (earliest after 14 days) (T2) was conducted.

**Fig. 1:**
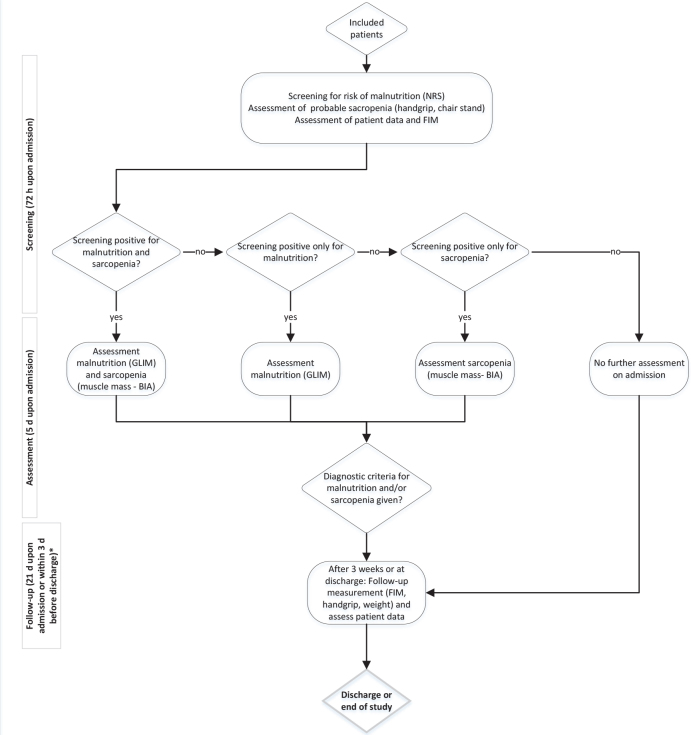
Patient flow, assessments, and measurements. BIA: Bioelectrical Impedance Analysis; CST: Chair Stand Test; GLIM: Global Leadership Initiative on Malnutrition; HGS: handgrip strength; NRS: Nutritional Risk Score 2002. *If discharge occurred first but at earliest 14 days after admission.

The patient flow and measurements are illustrated in Fig. 1.

### Primary outcome: prevalence of malnutrition and sarcopenia

Diagnosis of malnutrition was made upon positive risk screening. Risk of malnutrition was assessed by the Nutritional Risk Score (NRS 2002) (16). An NRS score of 3 or higher was considered as at risk of malnutrition.

The assessment for diagnosis of malnutrition was determined according to the Global Leadership Initiative on Malnutrition (GLIM) criteria by trained dietitians (17). Diagnosis of malnutrition was confirmed if at least 1 phenotypic (non-volitional weight loss, low BMI, reduced muscle mass) and 1 aetiologic (reduced food intake or assimilation, inflammation, or disease burden) criterion were met. Muscle mass was estimated based on the appendicular skeletal muscle mass index (ASMI) derived by BIA (BIA 101 BIVA Pro device, Akern Italy,) with classic tetrapolar technique (50 kHz). For the evaluation of appendicular skeletal muscle mass (ASMM), the Software BODYGRAM HBO (Version Akern, distributed by Fresenius Kabi AG, Bad Homburg, Germany) was used. Cut-off points for low ASMI, calculated from ASMM/height^2^, were < 7 kg/m^2^ for men and < 5.5 kg/m^2^ for women (15).

Sarcopenia was assessed according to the European Working Group on Sarcopenia in Older People (EWGSOP2) guidelines (8). However, due to the low sensitivity of the Sarcopenia Screening Questionnaire (SARC-F) (8), assessment of muscle strength by handgrip strength (HGS) and Chair Stand Test (CST) for the identification of probable sarcopenia was performed in all patients. Probable sarcopenia was identified when either HGS or CST was low. HGS was measured with the Martin Vigorimeter, balloon size 3 (Gebrüder Martin GmbH & Co. KG, Tuttlingen, Germany). Measurement was repeated 3 times at the dominant hand, recording the highest value. To define low HGS, cut-off points of < 50 kPa for men and < 34 kPa for women > 75 years old, and < 64 kPa for men and < 42 kPa for women ≤ 75 years old were applied (18). The CST was performed using the Five Times Sit-to-Stand Test. A cut-off value of ≥ 15 s or long-term inability to perform the test indicated probable sarcopenia (8).

In case of probable sarcopenia, muscle mass was assessed as mentioned above. Cut-off points for low ASMI were < 7 kg/m^2^ for men and < 5.5 kg/m^2^ for women (8).

Diagnosis of sarcopenia was confirmed when muscle strength and muscle mass were low (8).

### Secondary outcomes: Changes in nutritional and functional independence parameters between rehabilitation admission and discharge

Weight, HGS, and FIM were collected as follow-up parameters. The FIM is a measure of 18 activities of daily living, separated for motoric and cognitive items and frequently used in rehabilitation. The total FIM score ranges from 18–126 points with higher points reflecting better functionality.

The nutritional and physiotherapy interventions (time in min) and the duration of the rehabilitation stay were recorded to allow additional data analyses. Nutritional therapy was in our study defined as involvement of a dietitian by, e.g., group or individual counselling and education.

Data were collected and managed using REDCap electronic data capture tools (https://project-redcap.org/) hosted at the University of Applied Sciences and Arts Western Switzerland Wallis (19).

### Statistical analyses

A precision-based approach was used for the sample size calculation for the prevalence of malnutrition. To estimate a prevalence of 30–50% with a confidence interval width of less than 10%, 320 to 390 patients were required. The sample size was increased to 550 patients to obtain meaningful prevalence data for subgroup analysis (minimum 80 patients for each of the 6 rehabilitation disciplines). Patients who dropped out before the primary outcome assessment had to be replaced.

The study population was characterized using frequencies (*n*) and percentages (%) for categorical variables, and medians and 25th to 75th interquartile ranges (IQR) for continuous data.

The prevalence of malnutrition, sarcopenia, co-occurrence of both, as well as the risk of malnutrition and probable sarcopenia were reported as percentage (%) with 95% Wilson confidence interval (CI) by discipline. Additionally, patient characteristics were reported by malnutrition and sarcopenia status. Linear mixed models for HGS, weight, and FIM were fitted to the data with timepoint and either diagnosis of malnutrition or sarcopenia as main effects and a time:malnutrition or time:sarcopenia interaction effect respectively. For malnutrition, patients were grouped in a 3-level factor, namely “no risk”, “at risk but no confirmed diagnosis”, and “confirmed diagnosis”. For sarcopenia, patients were grouped in “no signs of sarcopenia”, “probable sarcopenia”, and “confirmed diagnosis”. All models were adjusted for age and sex. Site and ID-site interaction were included as random intercepts to take into account the nested structure of the data. Contrasts of interest – (within-group changes, between-group differences, and time-group interactions) – were computed from the fitted models. Residual analyses were performed to check model assumptions.

All analyses were performed using the R statistical software R version 4.3.1 (R Core Team, 2023; R Foundation for Statistical Compuring, Vienna, Austria), using the arsenal (20), lme4 (21), effects (22), and emmeans packages (23).

## Results

Overall, 558 patients were included in the study (see patient flow diagram in Fig. S1). Geriatric, pulmonary, and neurological rehabilitation were the disciplines with the most patients (*n* = 102, 100, and 100, respectively), followed by musculoskeletal (90), cardiovascular (87), and internal medicine/ oncological (79) rehabilitation (Table I).

**Table I:** Descriptive statistics of patient characteristics (overall and per discipline)

Characteristics	All (*n* = 558)	Geriatric (*n* = 102)	Pulmonary (*n* = 100)	Internal medicine/ oncological (*n* = 79)	Cardiovascular (*n* = 87)	Neurological (*n* = 100)	Musculoskeletal (*n* = 90)
General characteristics							
Age years, median (Q1, Q3)	73.0 (62.0, 80.0)	86.0 (80.2, 88.0)	70.0 (65.0, 76.0)	72.0 (65.0, 79.5)	66.0 (57.5, 76.0)	57.5 (48.0, 72.2)	75.0 (67.2, 80.0)
Sex male, *n* (%)	289 (51.8)	44 (43.1)	47 (47.0)	41 (51.9)	64 (73.6)	58 (58.0)	35 (38.9)
BMI kg/m^2^, median (Q1, Q3)	25.1 (22.2, 28.4)	24.0 (21.9, 27.6)	23.8 (20.2, 27.1)	24.8 (22.1, 28.0)	26.1 (23.2, 29.6)	25.4 (22.5, 27.8)	26.4 (24.1, 30.3)
Previous stay at ICU Yes, *n* (%)	177 (31.7)	21 (20.6)	39 (39.0)	32 (40.5)	57 (65.5)	24 (24.0)	4 (4.4)
Previous stay at ICU Unknown, *n* (%)	48 (8.6)	10 (9.8)	3 (3.0)	3 (3.8)	8 (9.2)	18 (18.0)	6 (6.7)
Polypharmacy, *n* (%)	493 (88.4)	98 (96.1)	78 (78.0)	73 (92.4)	83 (95.4)	81 (81.0)	80 (88.9)
Comorbidities							
Hypertension, *n* (%)	200 (35.8)	39 (38.2)	31 (31.0)	38 (48.1)	17 (19.5)	33 (33.0)	42 (46.7)
Coronary heart disease, *n* (%)	104 (18.6)	31 (30.4)	20 (20.0)	17 (21.5)	3 (3.4)	20 (20.0)	13 (14.4)
Acute respiratory tract infections, *n* (%)	34 (6.1)	12 (11.8)	1 (1.0)	13 (16.5)	4 (4.6)	3 (3.0)	1 (1.1)
Chronic respiratory disease, *n* (%)	57 (10.2)	17 (16.7)	1 (1.0)	17 (21.5)	7 (8.0)	5 (5.0)	10 (11.1)
Gastrointestinal disease, *n* (%)	52 (9.3)	17 (16.7)	4 (4.0)	13 (16.5)	3 (3.4)	7 (7.0)	8 (8.9)
Chronic renal failure, *n* (%)	86 (15.4)	28 (27.5)	17 (17.0)	13 (16.5)	12 (13.8)	6 (6.0)	10 (11.1)
Diabetes, *n* (%)	97 (17.4)	19 (18.6)	11 (11.0)	14 (17.7)	14 (16.1)	21 (21.0)	18 (20.0)
Osteoporosis, *n* (%)	57 (10.2)	20 (19.6)	11 (11.0)	6 (7.6)	2 (2.3)	5 (5.0)	13 (14.4)
Oncological diseases, *n* (%)	77 (13.8)	15 (14.7)	12 (12.0)	22 (27.8)	1 (1.1)	16 (16.0)	11 (12.2)
None of the above, *n* (%)	190 (34.1)	12 (11.8)	47 (47.0)	14 (17.7)	51 (58.6)	43 (43.0)	23 (25.6)
Number of comorbidities, median (Q1, Q3)	1.0 (1.0, 2.0)	2.0 (1.0, 3.0)	1.0 (1.0, 2.0)	2.0 (1.0, 3.0)	1.0 (1.0, 1.0)	1.0 (1.0, 2.0)	1.0 (1.0, 2.0)
Assessments							
FIM cognition score, median (Q1, Q3)	29.0 (26.0, 32.0)	29.0 (25.0, 31.0)	29.0 (26.8, 31.0)	30.0 (27.0, 34.0)	29.0 (28.0, 30.0)	25.5 (21.8, 29.0)	33.0 (29.0, 35.0)
FIM motor score, median (Q1, Q3)	64.0 (54.0, 72.0)	60.0 (47.2, 72.0)	66.5 (59.8, 71.0)	66.0 (57.0, 73.0)	68.0 (63.0, 72.5)	52.5 (35.2, 64.0)	66.5 (54.2, 74.8)
FIM total score, median (Q1, Q3)	93.0 (80.0, 102.0)	88.5 (71.0, 102.8)	96.0 (88.0, 101.0)	97.0 (83.5, 105.0)	97.0 (91.0, 102.0)	77.0 (59.2, 89.0)	99.0 (84.2, 108.0)

Q1, Q3: Interquartile ranges; BMI: body mass index; ICU: intensive care unit; FIM: Functional Independence Measure.

### Patient characteristics

All patient characteristics are displayed in Table I. Among all participants, 51.8% were male, the median age was 73.0 years, and median BMI 25.1 kg/m^2^. Polypharmacy was very common and occurred in 88.4% of patients. Previous ICU stay was reported for 31.7% and unknown for 8.6% of patients. Differences between the disciplines were observed with patients in geriatric rehabilitation being oldest (median 86.0 years) and patients in neurological rehabilitation youngest (median 57.5 years). The FIM total score medians ranged between 77.0 (Q1 59.2, Q3 89.0) points in neurological to 99.0 (Q1 84.2, Q3 108.0) points in musculoskeletal rehabilitation.

### Prevalence of malnutrition

**Fig. 2:**
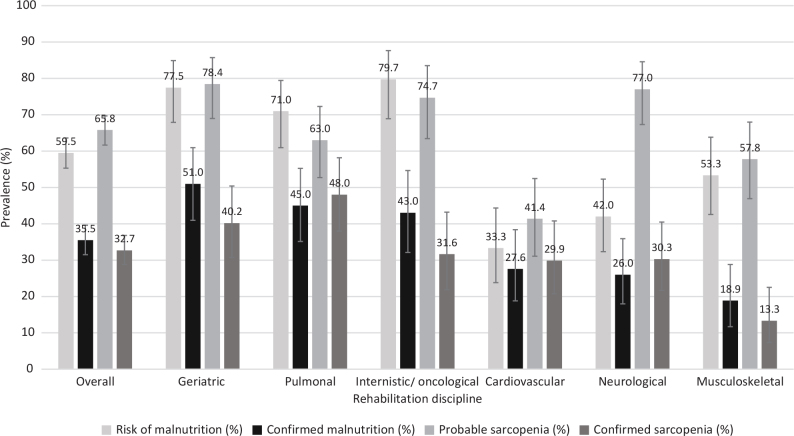
Prevalence of risk of malnutrition and sarcopenia and diagnosed malnutrition and sarcopenia according to rehabilitation discipline. Malnutrition screened with Nutritional Risk Score (NRS) 2002 and diagnosed according to Global Leadership Initiative on Malnutrition (GLIM) criteria, sarcopenia screened with handgrip strength (HGS) and Chair Stand Test (CST) and diagnosed according to EWGSOP2 criteria, data with mean and 95% confidence interval.

Depending on discipline, the risk of malnutrition ranged from 33.3% (95% CI 23.8, 44.4%) in cardiovascular patients to 79.7% (95% CI 68.9, 87.6%) in internal medicine/oncological patients (Fig. 2).

The overall prevalence of malnutrition in the study participants was 35.5% (95% CI 31.5, 39.6%) (Fig. 2). The prevalence rates were 51.0% (95% CI 40.9, 60.9%) for geriatric, 45.0% (95% CI 35.1, 55.2%) for pulmonary, 43.0% (95% CI 32.1, 54.6%) for internal medicine/oncological, 27.6% (95% CI 18.8, 38.4%) for cardiovascular, 26.0% (95% CI 18.0, 35.9%) for neurological, and 18.9% (95% CI 11.7, 28.8%) musculoskeletal rehabilitation, respectively (Fig. 2). Patients with diagnosis of malnutrition were older with a median age of 76.0 vs 69.0 years, had a lower BMI (22.4 kg/m^2^ vs 26.7 kg/m^2^) and a lower FIM total score (Table II). Sex and previous stay in the ICU were not different between these 2 groups.

**Table II:** Patient characteristics stratified by diagnosis of malnutrition and sarcopenia

Characteristics	Diagnosis malnutrition		Diagnosis sarcopenia	
	No (*n* = 360)	Yes (*n* = 198)	No (*n* = 375)*	Yes* (*n* = 182)
General characteristics				
Age, years, median (Q1, Q3)	69.0 (58.0, 79.0)	76.0 (69.0, 84.0)	71.0 (58.0, 79.5)	76.0 (68.0, 84.0)
Sex male, *n* (%)	185 (51.4)	104 (52.5)	188 (50.1)	101 (55.5)
BMI, kg/m^2^, median (Q1, Q3)	26.7 (23.9, 29.9)	22.4 (19.9, 25.3)	26.6 (23.9, 29.8)	22.2 (19.8, 24.9)
Previous stay at ICU Yes, *n* (%)	109 (30.3)	68 (34.3)	118 (31.5)	59 (32.4)
Previous stay at ICU Unknown, *n* (%)	34 (9.4)	14 (7.1)	36 (9.6)	11 (6.0)
Polypharmacy, *n* (%)	309 (85.8)	184 (92.9)	329 (87.7)	163 (89.6)
Comorbidities				
Hypertension, *n* (%)	138 (38.3)	62 (31.3)	153 (40.8)	47 (25.8)
Coronary heart disease, *n* (%)	55 (15.3)	49 (24.7)	65 (17.3)	39 (21.4)
Acute respiratory tract infections, *n* (%)	14 (3.9)	20 (10.1)	18 (4.8)	16 (8.8)
Chronic respiratory disease, *n* (%)	33 (9.2)	24 (12.1)	39 (10.4)	18 (9.9)
Gastrointestinal disease, *n* (%)	32 (8.9)	20 (10.1)	34 (9.1)	18 (9.9)
Chronic renal failure, *n* (%)	48 (13.3)	38 (19.2)	52 (13.9)	34 (18.7)
Diabetes, *n* (%)	59 (16.4)	38 (19.2)	61 (16.3)	36 (19.8)
Osteoporosis, *n* (%)	36 (10.0)	21 (10.6)	41 (10.9)	16 (8.8)
Oncological diseases, *n* (%)	45 (12.5)	32 (16.2)	49 (13.1)	28 (15.4)
None of the above, *n* (%)	129 (35.8)	61 (30.8)	123 (32.8)	66 (36.3)
Number of comorbidities, median (Q1, Q3)	1.0 (1.0, 2.0)	1.0 (1.0, 3.0)	1.0 (1.0, 2.0)	1.0 (1.0, 2.0)
Assessments				
FIM cognition score, median (Q1, Q3)	30.0 (27.0, 32.0)	28.0 (25.0, 31.0)	30.0 (27.0, 32.0)	28.0 (25.0, 30.0)
FIM motor score, median (Q1, Q3)	65.5 (55.0, 73.0)	62.5 (51.2, 70.0)	66.0 (55.0, 74.0)	61.0 (49.0, 69.0)
FIM total score, median (Q1, Q3)	95.0 (82.0, 104.0)	90.5 (76.0, 100.0)	95.0 (83.0, 105.0)	89.0 (73.0, 99.0)

* Total number of included patients *n* = 558, for sarcopenia diagnosis measurements 1 patient missing.

Values indicate median (interquartile ranges) for continuous and counts (proportions) for frequency data.

Q1, Q3: Interquartile ranges; BMI: body mass index; ICU: intensive care unit; FIM: Functional Independence Measure.

### Prevalence of sarcopenia

Probable sarcopenia ranged, depending on discipline, between 41.4% (95% CI 31.1, 52.4%) in cardiovascular patients and 78.4% (95% CI 69.0, 85.7%) in geriatric patients (Fig. 2).

The overall prevalence of confirmed sarcopenia was 32.7% (95% CI 28.8, 36.8%) (Fig. 2). Patients in pulmonary rehabilitation had the highest prevalence of sarcopenia of 48.0% (95% CI 38.0, 58.2%), followed by geriatric rehabilitation with 40.2% (95% CI 30.8, 50.4%), and internal medicine/oncological rehabilitation with 31.6% (95% CI 21.9, 43.2%) (Fig. 2). In cardiovascular and neurological rehabilitation, almost 1 in 3 patients was diagnosed with sarcopenia (29.9% and 30.3%, respectively), while in musculoskeletal rehabilitation, the prevalence was 13.3% (95% CI 7.4, 22.5%). Similar to the malnutrition diagnosis, patients with sarcopenia diagnosis had a higher median age (76.0 vs 71.0 years), a lower BMI (22.2 kg/m^2^ vs 26.6 kg/m^2^) and lower FIM cognition, motor, and total score than patients without diagnosis (Table II). Sex and previous stay in the ICU did not differ between patients with or without diagnosis.

### Co-occurrence of risk and diagnosis of malnutrition and sarcopenia

**Fig. 3:**
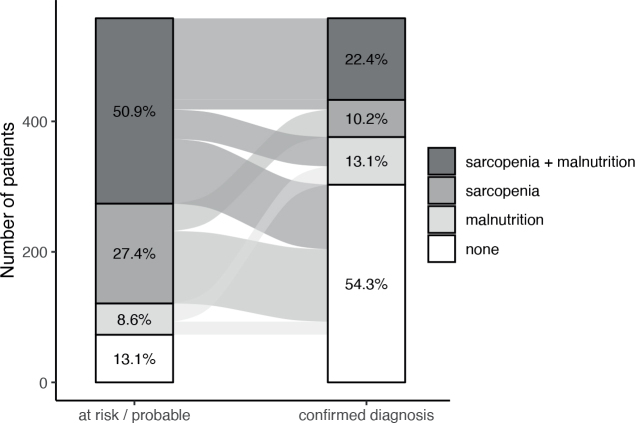
Co-occurrence of malnutrition and sarcopenia (%).

Co-occurrence of malnutrition and sarcopenia was observed in 22.4% of patients (Fig. 3). Diagnosed sarcopenia without risk of malnutrition was observed in 7.5% of patients and, equally, diagnosed malnutrition without probable sarcopenia in 7.5% of patients.

### Changes in muscle strength, bodyweight, and functional independence between rehabilitation admission and discharge

**Fig. 4:**
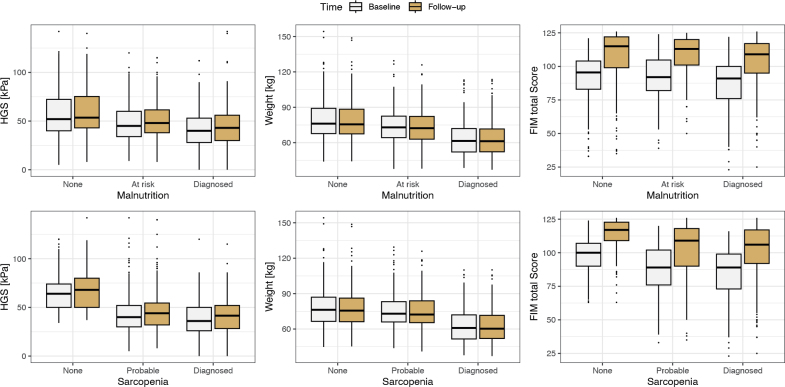
Changes in handgrip strength (HGS), bodyweight, and Functional Independence Measure (FIM) between admission and discharge in patients with no risk, risk of, and diagnosis of malnutrition and no risk, probable, and diagnosed sarcopenia. Descriptive statistics with mean and 95% confidence interval.

During rehabilitation on average, patients increased their HGS; patients with no malnutrition by 2.02 kPa (95% CI 0.86, 3.19), patients at risk by 3.08 kPa (95% CI 1.57, 4.58), and patients with diagnosed malnutrition by 3.82 kPa (95% CI 2.57, 5.07), respectively (Table III, Fig. 4). Patients at no risk and patients with a diagnosis of malnutrition did not change their weight between admission and discharge while patients at risk of malnutrition lost 1.14 kg (95% CI –1.64, –0.63) (Table III, Fig. 4). The FIM score did increase by 17.0 points (95% CI 14.9, 19.1) in patients at risk of malnutrition, by 16.8 points (95% CI 15.1, 18.5) in patients with diagnosed malnutrition, and by 14.8 points (95% CI 13.2, 16.4) in patients at no risk. In particular the FIM motor score increased (data not shown) and contributed most to the increase in total score. Both on admission and on discharge, malnourished patients still had a significantly lower HGS, weight, and FIM compared with patients without diagnosis (Table III, Fig. 4). Changes in HGS over the course of rehabilitation were higher in patients diagnosed with malnutrition compared with those at no risk of malnutrition (*p* < 0.01) and higher in patients with probable sarcopenia compared with no signs or diagnosed sarcopenia (*p* < 0.01). Patients at risk of malnutrition had a higher change in weight compared with the other groups (no risk, diagnosed) (*p* < 0.01). Changes in the FIM did not differ between groups for either malnutrition or sarcopenia.

**Table III:** Model-based estimated means of hand grip strength (HGS), bodyweight, and Functional Independence Measure (FIM) on admission and discharge in patients with no risk, risk of, and diagnosis of malnutrition and no risk, probable, and diagnosed sarcopenia, and changes according to diagnosis status

Status	HGS (kPa)			Weight (kg)			FIM		
	Admission	Discharge	Estimated change	Admission	Discharge	Estimated change	Admission	Discharge	Estimated change
	Estimated means [95% CI]			Estimated means [95% CI]			Estimated means [95% CI]		
No malnutrition (*n* = 226)	53.6 [47.7, 59.4]^a^	55.6 [49.8, 61.4]^a^	2.02 [0.86, 3.19]^a^	76.6 [72.9, 80.2]^a^	76.2 [72.6, 79.9]^a^	-0.31 [-0.71, 0.08]^a^	90.2 [71.6, 109]^a^	105.0 [86.4, 124]^a^	14.8 [13.2, 16.4]^a^
Risk of malnutrition (*n* = 134)	50.7 [44.8, 56.6]^a^	53.8 [47.9, 59.7]^a^	3.08 [1.57, 4.58]^a,d^	76.9 [73.0, 80.9]^a^	75.8 [71.9, 79.7]^a^	-1.14 [-1.64, -0.63]^b,d*^	86.9 [68.4, 105]^a,b^	103.9 [85.4, 122]^a,b^	17.0 [14.9, 19.1]^a^
Malnutrition (*n* = 198)	43.7 [37.9, 49.6]^b^	47.6 [41.7, 53.4]^b^	3.82 [2.57, 5.07]^d^	64.1 [60.4, 67.8]^b^	64.1 [60.4, 67.9]^b^	0.06 [-0.36, 0.47]^a^	83.5 [64.9, 102]^b^	100.3 [81.7, 119]^b^	16.8 [15.1, 18.5]^a^
No sarcopenia (*n* = 191)	63.1 [57.8, 68.4]^a^	64.8 [59.6, 70.1]^a^	1.73 [0.47, 2.70]^a^	75.5 [71.4, 79.6]^a^	75.1 [71.0, 79.2]^a^	-0.40 [-0.83, 0.03]^a^	91.8 [74.5, 109.1]^a^	107.9 [90.6, 125.2]^a^	16.1 [14.3, 17.9]^a^
Probable sarcopenia (*n* = 184)	46.6 [41.4, 51.9]^b^	50.2 [44.9, 55.4]^b^	3.54 [2.27, 4.82]^d^	78.2 [74.2, 82.3]^a^	77.7 [73.6, 81.7]^a^	-0.59 [-1.02, -0.15]^a^	87.0 [69.7, 104.3]^b^	102.5 [85.3, 119.8]^b,c^	15.5 [13.7, 17.3]^a^
Sarcopenia (*n* = 182)	39.2 [33.9, 44.4]^c^	42.6 [37.3, 47.8]^c^	3.39 [2.07, 4.71]^a,d^	62.8 [58.7, 66.9]^b^	62.7 [58.6, 66.8]^b^	-0.15 [-0.60, 0.30]^a^	82.6 [65.3, 99.8]^d^	99.0 [81.8, 116.3]^c^	16.4 [14.6, 18.2]^a^

Model-based estimated means and 95% CI on admission, discharge, and changes according to diagnosis status and derived from the linear-mixed models.

Pairwise contrasts tested between patients with no, risk of, or diagnosed malnutrition and, tested separately, with no, probable, and diagnosed sarcopenia at a given time point; different letters indicate significant differences between groups at a given time point (*p* < 0.01, except ^d^ *p* < 0.05), e.g., value for HGS at admission group no malnutrition is not significantly different (*p* < 0.01) from group risk for malnutrition (same letter ^a^) but both groups are different from group malnutrition (*p* < 0.01) (letter ^b^).

^b,d*^ contrast of changes in weight between no risk/risk of malnutrition *p* < 0.05 (^d^), contrast of changes in weight between risk/diagnosis of malnutrition *p* < 0.01 (^b^).

In patients with sarcopenia, similar change trends were observed. On average, all patients increased their HGS (Table III, Fig. 4). Weight remained stable in patients with no or diagnosed sarcopenia but decreased in patients with probable sarcopenia by 0.59 kg (95% CI –1.02, –0.15). The FIM increased on average in all patients. On discharge, sarcopenic patients had a lower HGS, weight, and FIM than patients without sarcopenia (*p* < 0.01).

### Nutritional counselling during rehabilitation

Around half of the of patients (49.1%) received nutritional therapy including education in the form of group counselling (Table IV). Approximately one-third of patients (37.3%) at risk of malnutrition and one-third of patients diagnosed with malnutrition (35.4%) received no nutritional therapy, while 27.4% received nutritional therapy for other reasons. Most patients who received nutritional counselling received up to 90 min of counselling over 3 weeks.

**Table IV:** Nutritional therapy (min) in patients with no, risk of, and diagnosed malnutrition and no, probable, and diagnosed sarcopenia

Minutes of nutritional therapy			
Factor	No indication	Probable/at risk	Diagnosed
Malnutrition	*n* = 226	*n* = 134	*n* = 198
None	164 (72.6%)	50 (37.3%)	70 (35.4%)
up to 90 min	36 (15.9%)	48 (35.8%)	74 (37.4%)
91–180 min	23 (10.2%)	30 (22.4%)	46 (23.2%)
> 180 min	3 (1.3%)	6 (4.5%)	8 (4.0%)
Sarcopenia	*n* = 191	*n* = 184	*n* = 182
None	102 (53.4%)	90 (48.9%)	91 (50.0%)
up to 90 min	46 (24.1%)	59 (32.1%)	53 (29.1%)
91–180 min	38 (19.9%)	29 (15.8%)	32 (17.6%)
> 180 min	5 (2.6%)	6 (3.3%)	6 (3.3%)

1 *n* (%).

Likewise, the majority of patients with probable sarcopenia (48.9%) and those with diagnosed sarcopenia (50.0%) did not receive nutritional counselling (Table IV). If counselling occurred, the duration was most often up to a total of 90 min (32.1% and 29.1%, respectively).

## DISCUSSION

Our multi-centre study showed a varying but overall high prevalence of malnutrition and sarcopenia in all rehabilitation disciplines, which requires clinical attention. While HGS and FIM increase during rehabilitation, changes are small and indicate that patients diagnosed with malnutrition and sarcopenia are discharged while still in a malnourished and/or sarcopenic state. Furthermore, bodyweight decreases in patients at risk of malnutrition, requiring adequate follow-up after discharge.

### Prevalence of malnutrition and sarcopenia

In general, variations in screening and assessment methods and definitions of malnutrition and sarcopenia pose challenges to comparing results from prevalence studies. In our study, using the screening tool NRS 2002, 60% of all patients and up to 80% in geriatric and internal medicine/oncological patients were found to be at risk of malnutrition. To our knowledge, no other study has analysed the prevalence of malnutrition across rehabilitation disciplines. The highest prevalence of malnutrition of 51% in our study was observed in the geriatric population and within previously reported ranges (12, 24–26). Age is a known risk factor for malnutrition (27). In our study, according to the Swiss rehabilitation classification, internal medicine and oncological patients were pooled and prevalence of malnutrition was high at 43%. Data in the acute hospital setting report a risk and prevalence of malnutrition in various cancer patients of between 24% and 51% (28–30).

Our prevalence data on malnutrition in pulmonary rehabilitation were 45.0% and thus similar to patients with COPD in pulmonary rehabilitation, categorized with a rate of 46% as malnourished (assessed by the PG-SGA) (31), while in another study in COPD patients, only 22.6% were malnourished assessed by GLIM criteria (32). Differences in degree of obstruction, besides differences in assessment method and patient population (not all patients in our study had COPD), might explain the variation in results.

Patients undergoing cardiovascular rehabilitation in our study had a prevalence of malnutrition of 27.6% and a risk of 33.3%, lower than in a prospective study in cardiac rehabilitation with a risk of malnutrition of 42%, measured by Mini Nutritional Assessment (33). Neurological patients had a prevalence of malnutrition of 26.0%, comparable to a study in patients after acute stroke (34).

The prevalence of probable (74%) and confirmed (40.2%) sarcopenia in geriatric patients in our study was substantially higher than the prevalence found in a Swiss cohort of geriatric acute and rehabilitation patients (24.6% and 22.6% respectively) (11) or an Australian study of geriatric rehabilitation patients (19%) (12). While we defined probable sarcopenia as either reduced HGS or CST, the other studies identified patients at risk solely on the basis of low HGS (11,12). In our study, 51 geriatric patients had a low HGS and 72 a low CST (data not shown). This could explain higher numbers of probable and diagnosed sarcopenia. Other studies found that screening with CST identified up to twice as many cases of probable sarcopenia compared with HGS (35,36). According to the EWGSOP2 criteria, HGS and CST are recommended to identify low muscle strength (8). Our study confirms that both measures are valuable to identify patients with low muscle strength.

Studies in other disciplines with varying methods showed a somewhat higher prevalence of sarcopenia of 56% (95% CI 0.46–0.65) (5 studies after hip fracture and 1 after general deconditioning) (10) and 47.9% (older neurological rehabilitation patients) (37), potentially due to different patient populations, disease severity, or measurement cut-offs.

The co-occurrence of malnutrition and sarcopenia at 32.4% in geriatric rehabilitation patients in our study was higher than in geriatric rehabilitation patients in other studies, ranging from 13–23.5% (12, 38, 39), potentially resulting from different thresholds and using HGS solely for the assessment of probable sarcopenia.

### Changes in weight, HGS, and FIM during rehabilitation

While in our study bodyweight was stable in patients not at risk and those with a diagnosis of malnutrition, it decreased slightly in those at risk of malnutrition. The small difference of on average 1.1 kg (95% CI –1.64;–0.63) suggests a risk that patients continue to lose weight and become malnourished after discharge. Other studies emphasize the importance of targeting patients at risk of malnutrition by early nutritional interventions (3, 40). In a meta-analysis, the risk of mortality increased in adults ≥ 65 years of age and a BMI < 22, indicating that, in particular for older adults, weight management and reduction of underweight/low weight is of high importance (41).

Handgrip strength is a supportive measure in the determination of malnutrition, a widely accepted simple and reliable measurement associated with nutritional status, and reduced HGS is an indicator of increased postoperative complications, increased length of hospitalization, higher re-hospitalization rate, and decreased physical status (42–44). It is also considered a sensitive parameter and reacts faster to metabolic changes compared with bodyweight (42). An inverse association between HGS and malnutrition was shown in some studies (45, 46). In our study, HGS increased on average in all patients during rehabilitation. However, the changes were small and of limited clinical relevance, but in line with previous studies with interventions over a similar short time period (45).

The functional independence measure, in particular the motor score, increased on average in all patients in our study during rehabilitation but remained lower in patients diagnosed with malnutrition and/or sarcopenia. This finding is in agreement with studies showing lower FIM values in patients with sarcopenia on rehabilitation discharge compared with patients without (47–49). Furthermore, an inverse association of sarcopenia with FIM on discharge, worse recovery of FIM during rehabilitation, and higher mortality in sarcopenic patients was shown (49).

In our study, while almost all patients received a physiotherapy intervention (97.3%, data not shown) during rehabilitation, in only 49.1% has a dietitian been involved in the rehabilitation programme. It is possible that patients received nutritional interventions such as prescription of enriched meals and/or oral nutritional supplements without consultation with a dietitian. However, this high rate shows that stronger emphasis should be placed on institutional processes for nutritional management to prevent malnutrition. A study in France showed that only 57.6% of malnourished cancer patients received nutritional support (29), confirming the lacking nutritional intervention of our study. A recent study investigated nutritional care practices in geriatric rehabilitation across Europe (50). According to the survey, in Central Europe 98% of respondents indicated that they had an individual dedicated to nutritional care but only 67% indicated that the individual has sufficient time to dedicate to patients, mainly due to shortage of staff to perform screening and nutritional interventions.

Raised awareness among health professionals, adequate training, and availability of equipment to perform the measurements, institutional support, interprofessional collaboration, and financial reimbursement are important factors to prevent lack of diagnosis and lack of interventions for malnutrition and sarcopenia and should be enforced (51, 52). Assessment of the Global Malnutrition Composite Score could support institutions to adequately screen for malnutrition, plan therapeutic interventions, and document the findings (53).

Our study has several limitations. While severe dehydration or hyperhydration have been established as exclusion criteria to prevent bias in muscle mass and bodyweight assessments, the potential for overhydration in some patients remains a concern. The lack of easily applicable methods and clear thresholds complicates the accurate determination of overhydration and could have led to underestimation of muscle mass and overestimation of bodyweight and BMI. Evaluation of bioimpedance vector analysis would have been useful to reduce this potential bias. We did not gather comprehensive details on diagnoses and other disease-specific characteristics, potentially limiting the external validity of our study cohort. We also did not capture nutritional intake and nutritional interventions other than counselling to further explain the effects on weight and muscle strength.

The investigators identified further research needs. The long-term follow-up of malnourished and/or sarcopenic patients pre- and post-rehabilitation could help to improve patient care. Institutional needs and opportunities to improve nutritional care in various rehabilitation settings are another research area of interest. While physiotherapy is regularly prescribed in rehabilitation, nutrition therapy has had a lower priority so far, although it should go hand in hand with physiotherapy interventions for optimal care in patients at risk of malnutrition or sarcopenia.

In conclusion, our study underscores the notable prevalence of malnutrition and sarcopenia among patients undergoing inpatient rehabilitation. Across various rehabilitation disciplines, nearly half of all patients are diagnosed with malnutrition, sarcopenia, or both. Even after successful rehabilitation, a substantial number of patients are discharged with low bodyweight and low values of HGS and FIM. Consequently, suitable screening methods and nutritional interventions should be incorporated into the routine practices of rehabilitation clinics to proactively address and mitigate further nutritional and muscular decline. Continued long-term individual nutritional support after discharge is required to combat negative health consequences.

## Supplementary Material

MALNUTRITION AND SARCOPENIA IN INPATIENT REHABILITATION: PREVALENCE AND ASSOCIATIONS WITH CHANGES IN BODYWEIGHT, MUSCLE STRENGTH, AND FUNCTIONAL INDEPENDENCE

## Trial details

The study was registered at ClinicalTrials.gov with the registration number NCT05639985.
