# Supplementary figures and images for: MALNUTRITION AND SARCOPENIA IN INPATIENT REHABILITATION: PREVALENCE AND ASSOCIATIONS WITH CHANGES IN BODYWEIGHT, MUSCLE STRENGTH, AND FUNCTIONAL INDEPENDENCE
Source: J Rehabil Med. 2025 Feb 25;57:42215. doi: 10.2340/jrm.v57.42215 (PMC11877858; doi:10.2340/jrm.v57.42215)

Fig. S1. Recruitment flow diagram

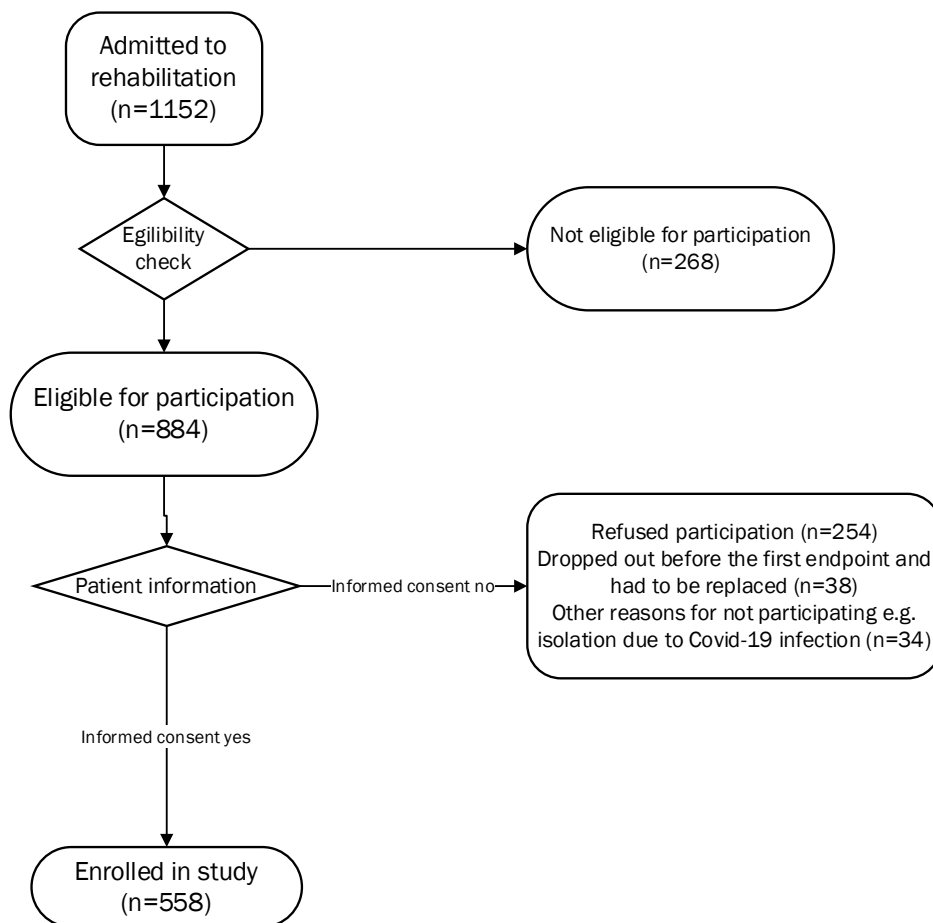

Supplement: MALNUTRITION AND SARCOPENIA IN INPATIENT REHABILITATION: PREVALENCE AND ASSOCIATIONS WITH CHANGES IN BODYWEIGHT, MUSCLE STRENGTH, AND FUNCTIONAL INDEPENDENCE [file JRM-57-42215-s1.pdf]
